# Overexpression of CCNE1 confers a poorer prognosis in triple-negative breast cancer identified by bioinformatic analysis

**DOI:** 10.1186/s12957-021-02200-x

**Published:** 2021-03-23

**Authors:** Qianqian Yuan, Lewei Zheng, Yiqin Liao, Gaosong Wu

**Affiliations:** Department of Thyroid and Breast Surgery, Zhongnan Hospital of Wuhan University, 169 Donghu Road, Wuhan, Hubei 430071 China

**Keywords:** Triple-negative breast cancer, GEO, CCNE1, Prognosis

## Abstract

**Background:**

Triple-negative breast cancer (TNBC) is a major subtype of breast cancer. Due to the lack of effective therapeutic targets, the prognosis is poor. In order to find an effective target, despite many efforts, the molecular mechanisms of TNBC are still not well understood which remain to be a profound clinical challenge.

**Methods:**

To identify the candidate genes in the carcinogenesis and progression of TNBC, microarray datasets GSE36693 and GSE65216 were downloaded from the Gene Expression Omnibus (GEO) database. The differentially expressed genes (DEGs) were identified, and functional and pathway enrichment analyses were performed using the Gene Ontology (GO) and Kyoto Encyclopedia of Genes and Genomes (KEGG) databases via DAVID. We constructed the protein-protein interaction network (PPI) and performed the module analysis using STRING and Cytoscape. Then, we reanalyzed the selected DEG genes, and the survival analysis was performed using cBioportal.

**Results:**

A total of 140 DEGs were identified, consisting of 69 upregulated genes and 71 downregulated genes. Three hub genes were upregulated among the selected genes from PPI, and biological process analysis uncovered the fact that these genes were mainly enriched in p53 pathway and the pathways in cancer. Survival analysis showed that only CCNE1 may be involved in the carcinogenesis, invasion, or recurrence of TNBC. The expression levels of CCNE1 were significantly higher in TNBC cells than non-TNBC cells that were detected by qRT-PCR (*P* < 0.05).

**Conclusion:**

CCNE1 could confer a poorer prognosis in TNBC identified by bioinformatic analysis and plays key roles in the progression of TNBC which may contribute potential targets for the diagnosis, treatment, and prognosis assessment of TNBC.

**Supplementary Information:**

The online version contains supplementary material available at 10.1186/s12957-021-02200-x.

## Introduction

Breast cancer (BC) is considered the most commonly diagnosed malignant tumor and the leading cause of cancer-related death among females worldwide [1]. Triple-negative breast cancer (TNBC) is one of the main tumor subtypes of BC, which lacks the expression of hormone receptors (estrogen/progesterone, ER/PR) and human epidermal growth factor receptor 2 (HER2) amplification, leading to a lack of effective treatment against the corresponding targets or receptors in addition to chemotherapy and radiotherapy. It accounts for 10–15% of all BC [2], and it usually appears in the form of high-grade invasive ductal carcinoma and has a higher early recurrence rate of between the first and third year of treatment, with the majority of deaths occurring within the first 5 years [3]. And it often manifests as distant metastases and is associated with poorer prognosis with comparison to other breast cancer subtypes [4, 5].

Due to the limited understanding of the pathogenesis, development, reproduction, and molecular mechanisms of TNBC, the mortality rate remains high. Therefore, it is of great importance to illuminate the exact molecular mechanisms of carcinogenesis, proliferation, and recurrence of TNBC in order to further develop more efficient diagnostic means as well as therapeutic options. In recent years, there have been some bioinformatic studies on TNBC, which can help identify the differentially expressed genes (DEGs) associated with TNBC carcinogenesis and progression and the functional pathways in which these genes participate, proving that integrated bioinformatical approaches is helpful for us to better explore the potential mechanisms. Changes in expression levels can often reflect pathological conditions, and proteins encoded by these DEGs may be involved in different biological processes, cellular behaviors, and molecular pathways during tumor progressions. In view of the diversity and complexity of TNBCs, omics technologies are necessary instruments to expand our understanding of TNBC subtypes [6]. As biomarkers, PR, ER, and ERBB2/HER2 can be used as prognostic targets of breast carcinoma and are helpful to suggest the most appropriate chemotherapeutic treatments. BRCA1 and BRCA2 gene mutations and homologous recombination (HR) pathways are involved in the DNA damage repair in TNBC [7].

Transcriptomic analysis showed that different genes such as MKI67, TOP2A, and EGFR were overexpressed in TNBC, providing different treatment directions for TNBC subtypes [6]. In this study, two microarray datasets (GSE36693 and GSE65216) containing both TNBC and non-TNBC samples were retrieved from Gene Expression Omnibus (GEO) and downloaded for further acquiring DEGs. The DEGs in the two datasets above were obtained using the GEO2R online tool in their respective data sets and then using the Venn chart software to obtain the intersection. The Database for Annotation, Visualization, and Integrated Discovery (DAVID) was used to analyze these DEGs including Gene Ontology (GO) and Kyoto Encyclopedia of Genes and Genomes (KEGG) pathways, the former part containing biological process (BP), cellular component (CC), and molecular function (MF). We constructed protein-protein interaction (PPI) network using STRING (Search Tool for the Retrieval of Interacting Genes), a convenient online software, and then applied Cytoscape as well as Molecular Complex Detection (MCODE) for extra analysis of DEGs in order to identify some hub genes. Subsequently, re-analysis of important genes by GO and KEGG pathway enrichment was performed. Moreover, Gene Expression Profiling Interactive Analysis (GEPIA) was utilized to further validate the DEG expression between TNBC tissues and non-TNBC tissues. Then, the survival of hub genes was analyzed by cBioPortal.

## Materials and methods

### Dataset selection

NCBI-GEO is considered as a public dataset containing countless microarray information [8], from which the gene expression profile of GSE36693 and GSE65216 in TNBC and non-TNBC tissues was downloaded. And the GSE36693 dataset contained 21 TNBC and 66 non-TNBC samples. GSE65216 contained 55 TNBC and 109 non-TNBC samples.

### Data processing and identification of DEGs

DEGs between TNBC samples and non-TNBC specimen were identified via GEO2R website (http://www.ncbi.nlm.nih.gov/geo/geo2r) with cutoff levels of log2 (fold-change) > 2 and adjusted *P* value <0.01 [9]. Then, through the Venn software, the intersection of the unsettled data in TXT format was taken and graphed online to identify common DEGs in the two databases.

### GO and KEGG enrichment analyses of DEGs

GO analysis is a widely used approach to make a definition of genes and their downstream product to identify unique biological properties of high-throughput information [10]. KEGG is a collection of databases that manage genomes, diseases, biological pathways, and relative chemical materials [11]. And the online tool, DAVID (http://david.ncifcrf.gov), is utilized to integrate biological data and provides a complete series of functional annotation information of genes and their proteins for researchers to obtain biological data [12]. In this study, DAVID was used to perform enrichment analysis of DEG’s BP, MF, CC, and its pathways (*P* < 0.05).

### PPI network and module analysis

The online tool STRING (http://string-db.org) can be used to construct a PPI network [13]. Then, the results of STRING were further analyzed and visualized by Cytoscape to explore the possible association of these DEGs, with maximum number of interactors = 0 and confidence level≥0.4 [14]. The MCODE of Cytoscape is an APP based on topological structure for clustering some known networks to find areas of their dense connectivity for further bioinformatic uses [15]. Thus, MCODE was utilized to check modules of the PPI network (MCODE scores>5, degree cutoff=2, max. depth=100, k-core=2, and node score cutoff=0.2).

### RNA sequencing expression of core genes

GEPIA is a network tool for cancer and normal gene expression profiling and interactive analyses to conduct differential expression analysis and survival analysis of genes of interest [16]. GEPIA website was applied to perform RNA expression analysis on these DEGs through TCGA data samples to verify.

### Survival analysis

The cBioPortal online platform provides a visual analysis instrument for interactive exploration of diverse cancer genome datasets [17, 18], allowing users to perform survival analysis based on DNA mutation data and CNA data, and visually displaying the patient’s overall survival (OS) and distant free survival (DFS) results in the form of Kaplan-Meier diagrams [19]. Kaplan-Meier curve was drawn through the cBioPortal online platform to analyze the OS of hub genes. We then calculate the hazard ratio and log rank *P* value with 95% confidence interval and display it in the figure.

### Experimental verification of the prognostic signature

Quantitative real-time polymerase chain reaction (qRT-PCR) was conducted in triplicate. β-Actin was used as internal control, and the 2^−ΔΔCt^ values were normalized to its levels. The primer sequences for qRT-PCR used in this study are shown in Supplementary Table 1.

## Results

### Identification of DEGs in TNBC

**Fig. 1:**
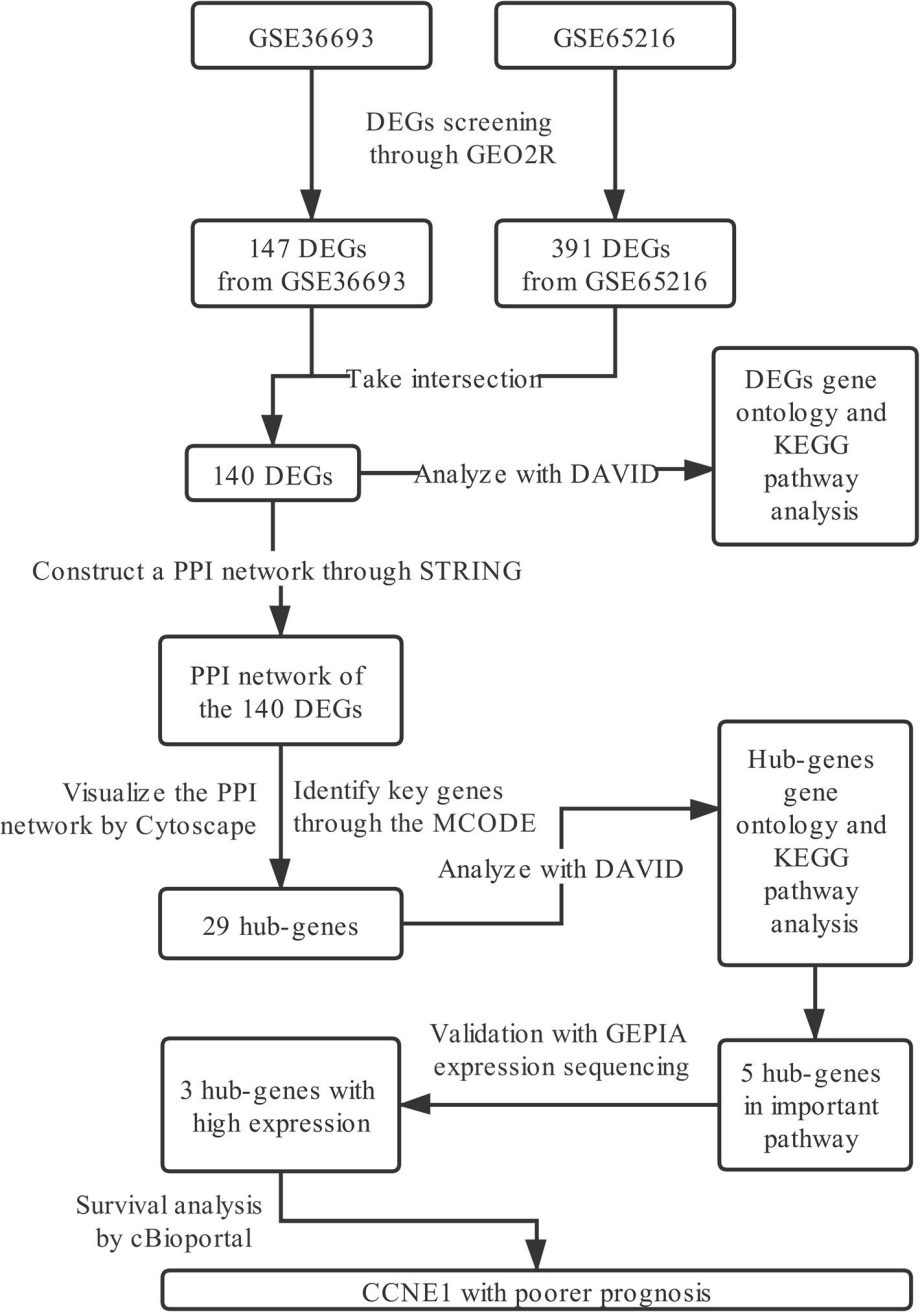
Flow diagram of the data selection

**Fig. 2:**
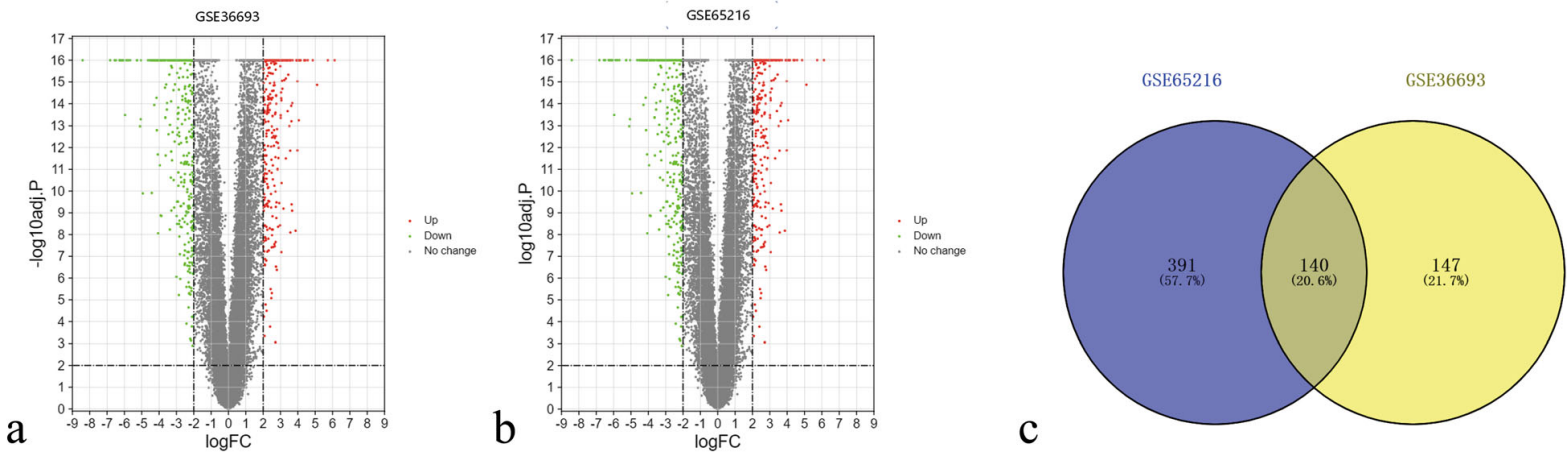
Volcano plots for DEGs in TNBC and non-TNBC tissues based on data from the GEO datasets. **a:** GSE36693, **b:** GSE65216. **c:** Take the intersection of the DEGs in the two data sets (GSE36693 and GSE65216) via the Venn diagram online tool, and get 140 common genes. Different colors in the figure mean different data sets

Figure 1 presented the workflow of this study. There were 76 TNBC tissues and 175 non-TNBC tissues in the current research. One hundred forty-seven and 391 DEGs from GSE36693 and GSE65216 were respectively extracted by GEO2R online tools. One hundred fifty-seven upregulated and 130 downregulated DEGs were conducted in GSE36693 (Fig. 2a) with the criteria of |logFC| > 2 and adjusted *P*-value < 0.05; 282 upregulated and 249 downregulated DEGs were gained in GSE65216 (Fig. 2b). Then, the Venn diagram online tool was utilized to intersect the DEGs in the two datasets and visualize them to identify the common DEGs. Consequences were that totally 140 common DEGs were detected in the TNBC tissues, including 69 downregulated genes with the limitation of logFC<−2 and 71 upregulated genes with the limitation of logFC > 2 (Fig. 2c).

### DEGs GO and KEGG pathway analysis in TNBC

All of the 140 DEGs were included by the DAVID online tool, and the results of GO analysis indicated that (1) as for BP, those upregulated genes were mainly enriched in the peripheral nervous system development, epidermis development, single organismal cell-cell adhesion, positive regulation of transcription from RNA polymerase II promoter, cytoskeleton organization etc., while downregulated DEGs were enriched in phosphatidylinositol 3-kinase signaling, positive regulation of transcription from RNA polymerase II promoter, regulation of intracellular transport, wound healing, and so on; (2) in the aspect of MF, upregulated DEGs were enriched in transcription factor activity, RNA polymerase II distal enhancer sequence-specific binding, chitinase activity, and chitin binding, and downregulated DEGs were significantly enriched in RNA polymerase II transcription factor binding, calcium ion binding, estrogen response element binding, dystroglycan binding, transcription regulatory region DNA binding; (3) for CC, upregulated DEGs were notably enriched in the extracellular space, extracellular exosome, epidermal lamellar body, and intermediate filament while downregulated genes were mainly enriched in extracellular space (Supplementary Table 2).

**Fig. 3:**
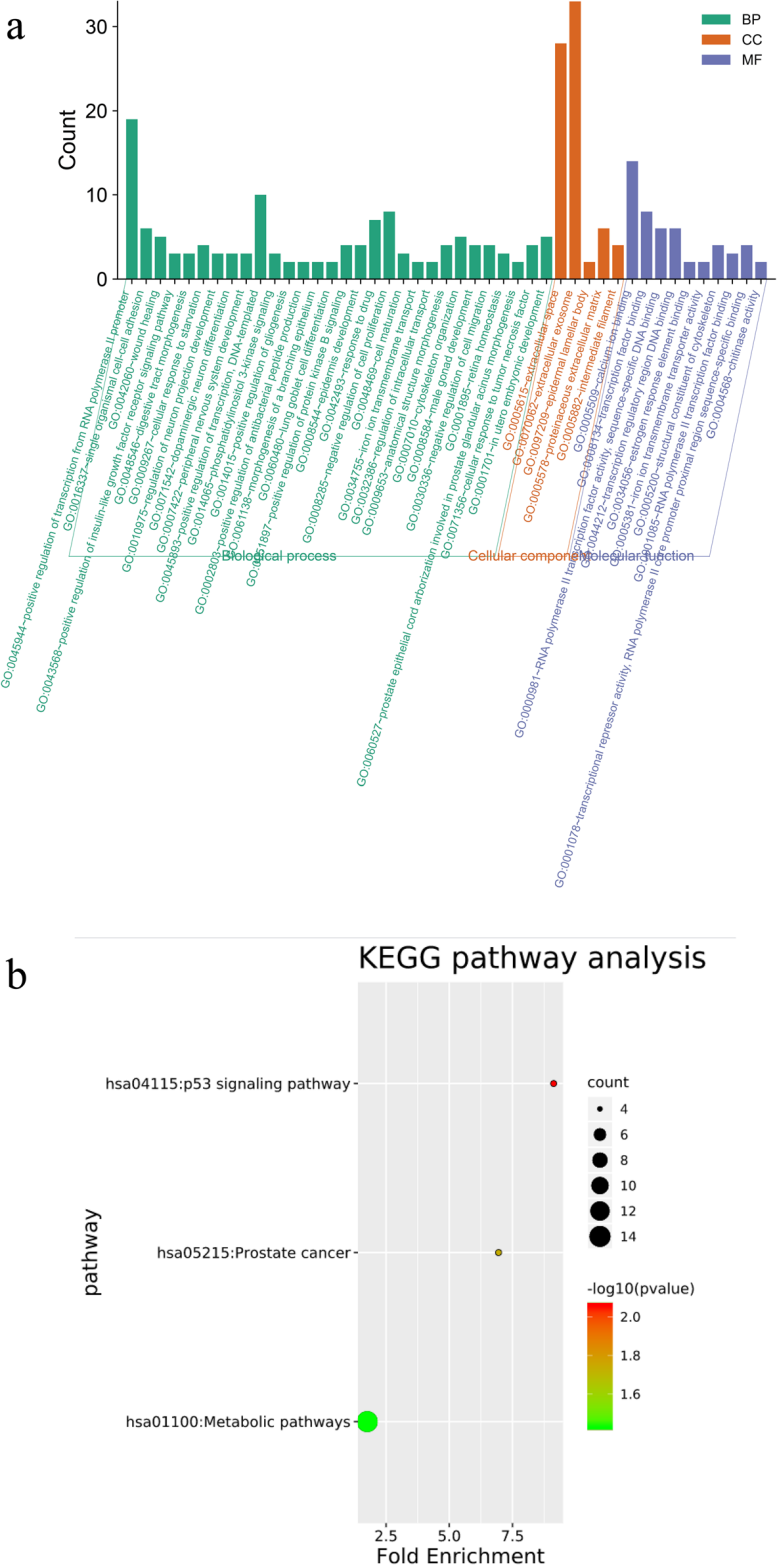
DAVID analysis of DEGs. **a:** GO functional annotation of top 45 enrichment terms. **b:** KEGG pathway enrichment analysis of top three enrichment terms. The count of genes enriched in terms is indicated by the node size; the *P* value is shown by the color, the redder the color, the more significant it is. DAVID, Database for Annotation, Visualization, and Integrated Discovery; DEGs, differentially expressed genes; GO, gene ontology; KEGG, Kyoto Encyclopedia of Genes and Genomes

Outcome of KEGG analysis is shown in Supplementary Table 3 and Fig. 3b, indicating that DEGs were significantly enriched in p53 signaling pathway, prostate cancer, and metabolic pathways (*P* < 0.05).

### Protein–protein interaction network (PPI) analysis

**Fig. 4:**
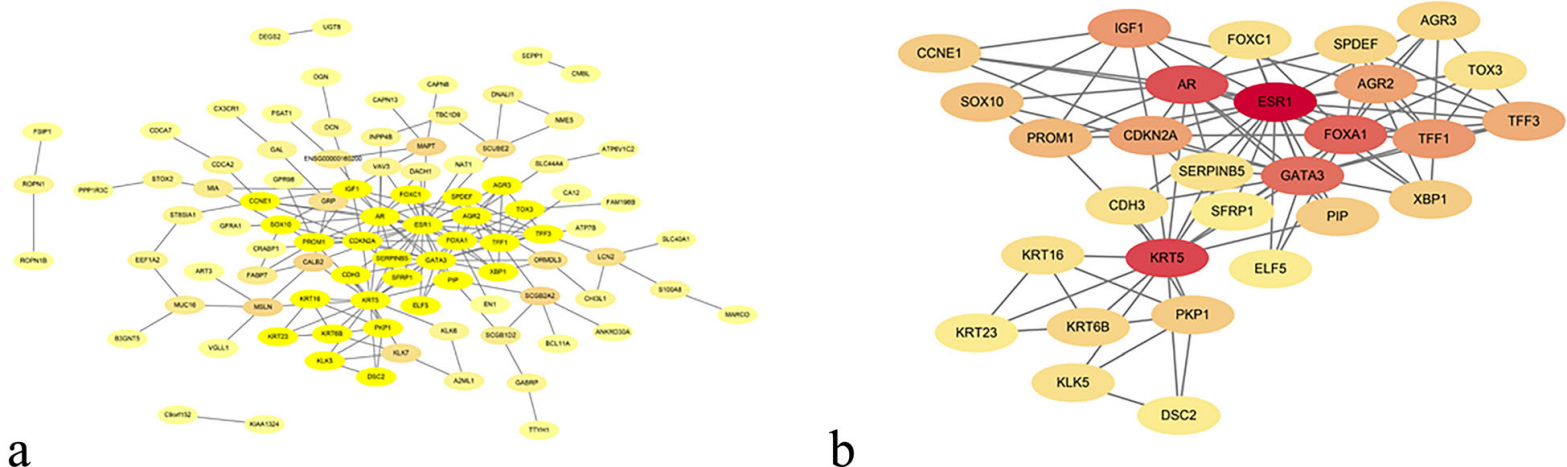
Common DEG PPI network set up by STRING database and module analysis. **a:** A total of 140 DEGs were used to construct a DEG PPI network complex, and 46 isolated nodes were removed. The nodes meant proteins; the edges meant the interaction of proteins. **b:** Module analysis via the Cytoscape software selected 29 hub genes (degree cutoff=2, node score cutoff=0.2, k-core= 2, and max. depth= 100)

The 140 DEGs including 69 upregulated genes and 71 downregulated genes were imported into the DEG PPI network complex which contained 94 nodes and 180 edges (Fig. 4a). And 46 isolated nodes were excluded. Then, Cytoscape MCODE was applied, and 29 central nodes were identified of the 94 nodes (Fig. 4b).

### Re-analysis of 29 selected genes by GO and KEGG pathway enrichment

**Fig. 5:**
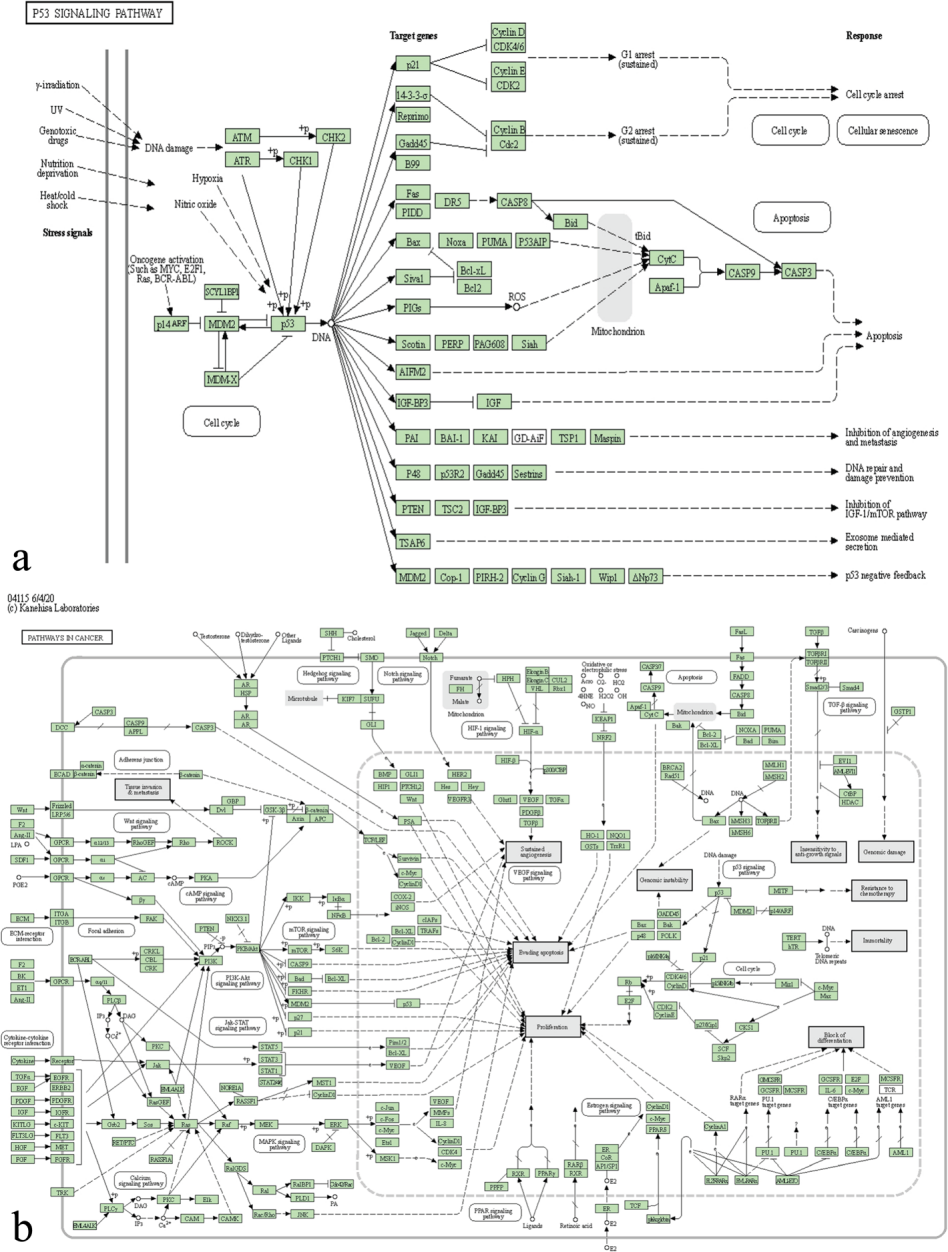
Re-analysis of 29 selected genes by KEGG pathway enrichment. **a:** Schematic diagram of p53 signaling pathway. **b:** Schematic diagram of pathways in cancer. Cyclin E means CCNE1. CycB means CCNB1

For the sake of figuring out the possible pathways of the 29 selected DEGs and verify whether they were consistent with the result of 140 DEG enrichment analysis, the GO and KEGG pathway enrichment analysis was performed on these 29 DEGs again by DAVID (*P* < 0.05). Outcome revealed that the 29 selected DEGs were evidently enriched in the p53 signaling pathway, pathways in cancer, oocyte meiosis, and prostate cancer (*P* < 0.05, Supplementary Tables 4 and 5 and Fig. 5).

### Analysis of core genes by the GEPIA

**Fig. 6:**
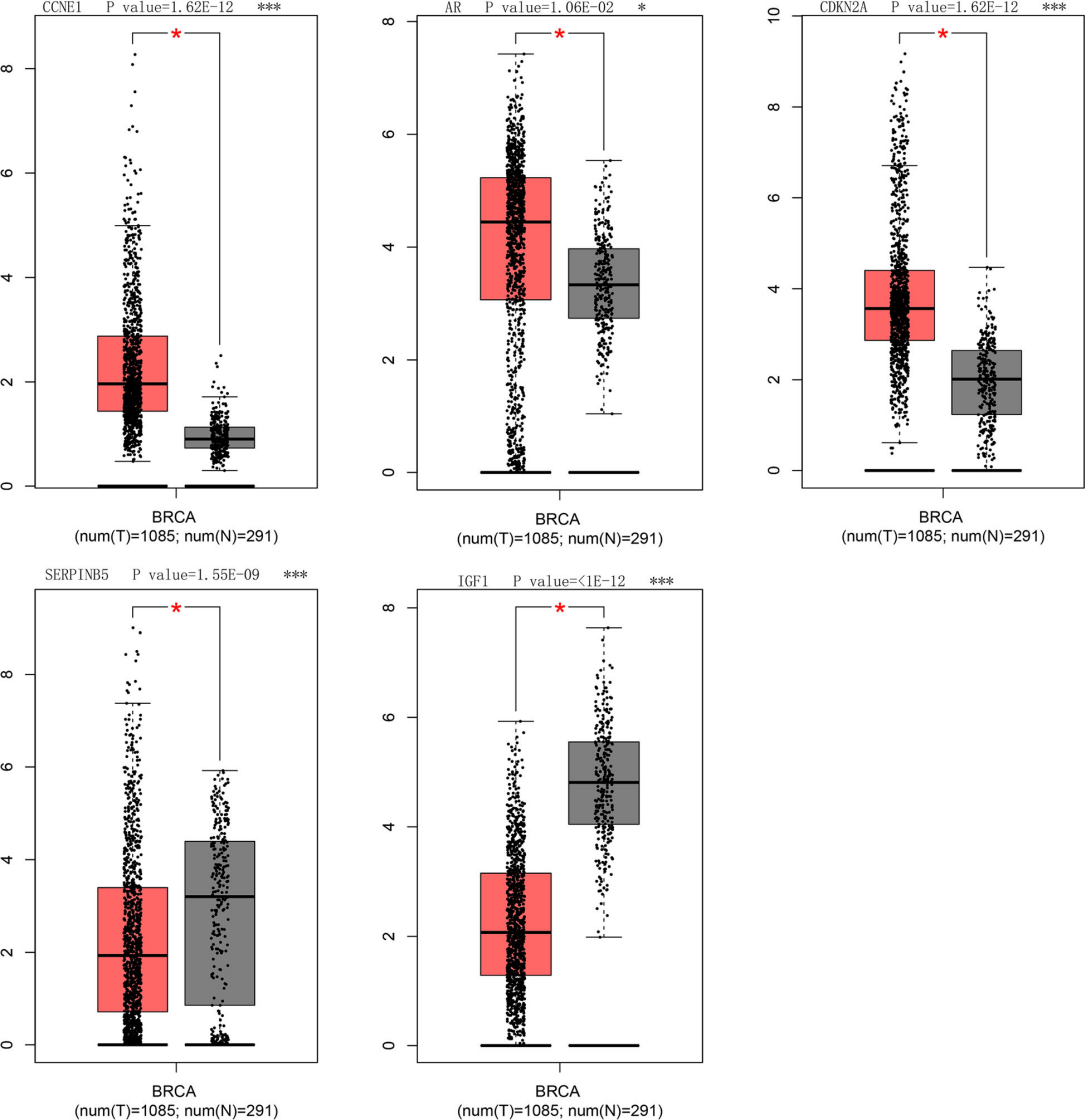
Significantly expressed five genes in TNBC samples compared to TNBC samples. Three of five genes had notable high expression in TNBC specimen compared to normal specimen (*P < 0.05). Red color refers to tumor tissues and blue color refers to normal samples

After taking the results of both PPI analysis and the KEGG pathway enrichment into consideration, we found that CCNE1, CDKN2A, AR, SERPINB5, and IGF1 among the 29 selected genes could play a key role in common significantly enriched pathways. Then, GEPIA was utilized to analyze the differences in the expression of these 5 genes of TNBC and non-TNBC tissues. Compared with non-TBNC samples, three hub genes (CCNE1, AR, CDKN2A) were highly expressed while two genes (SERPINB5, IGF1) were not in TNBC samples (*P* < 0.05, Fig. 6).

### Survival analysis using cBioportal

**Fig. 7:**
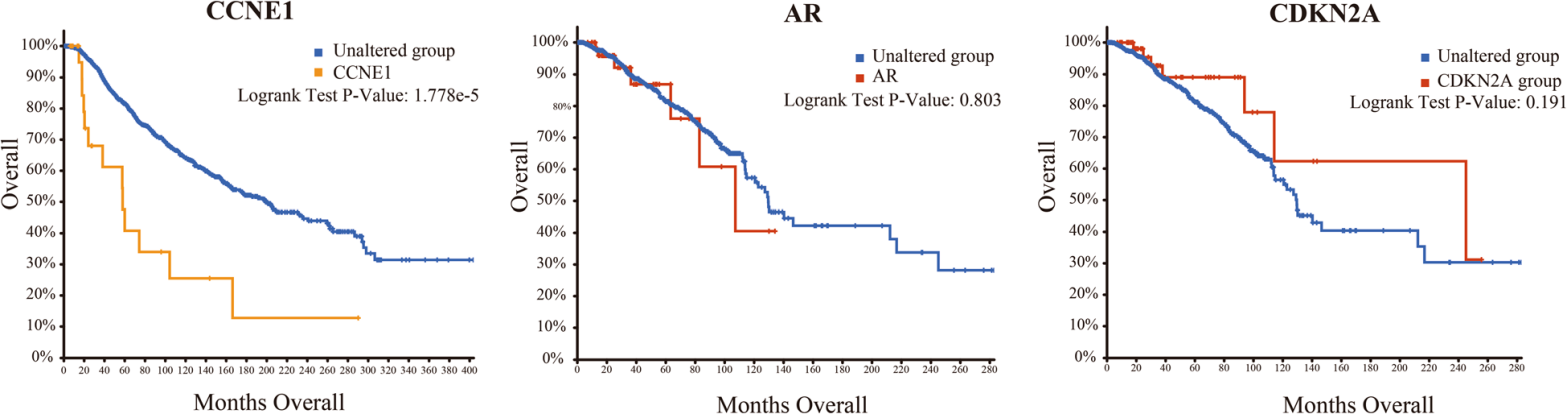
The prognostic information of the three hub genes. cBioportal was utilized to identify the prognostic information of the three core genes; CCNE1 had a significantly worse survival rate (*P* < 0.05)

cBioportal was used to identify 3 hub genes using survival data. Only CCNE1 had an obviously worse survival, while the other two genes did not (*P* < 0.05, Fig. 7).

### qRT-PCR analysis

**Fig. 8:**
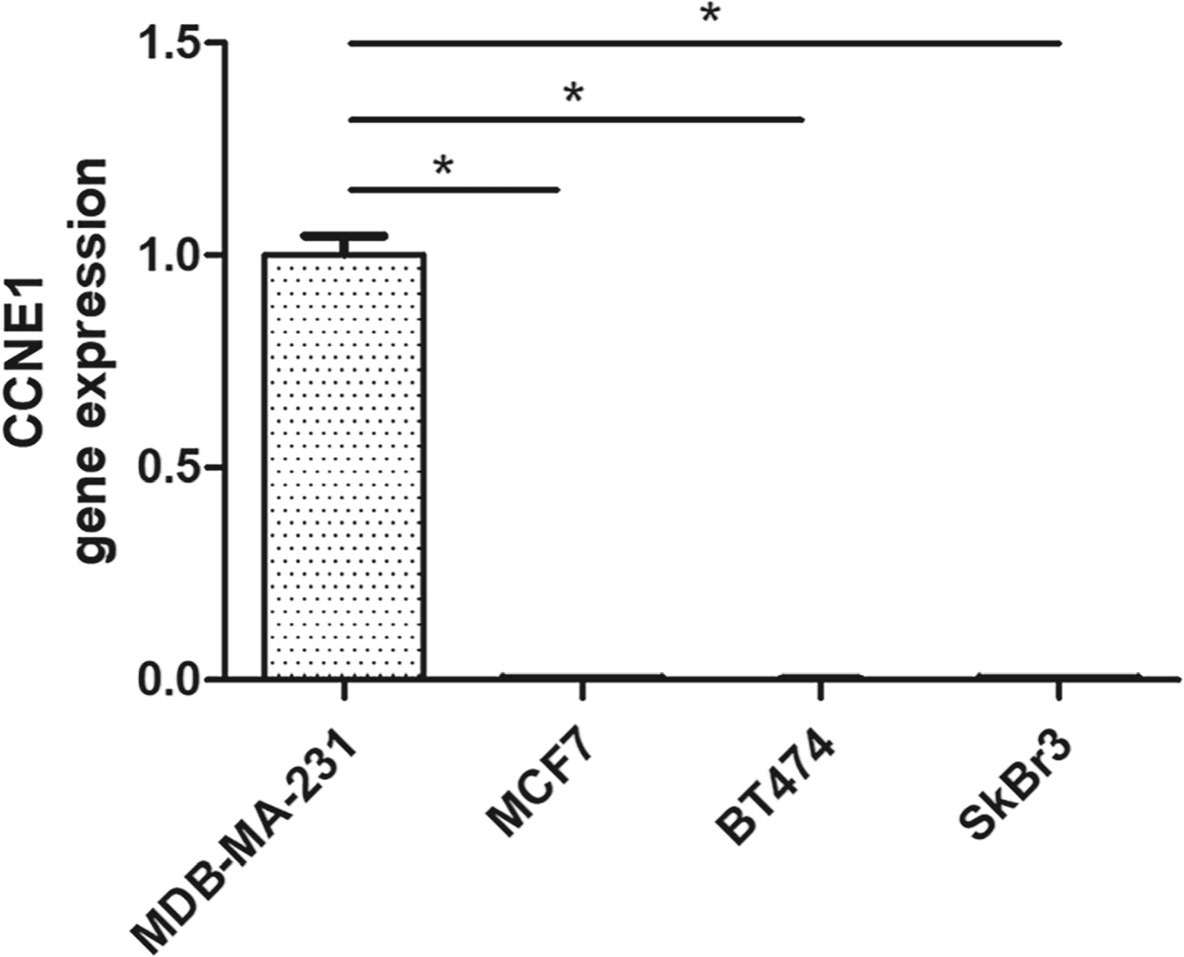
CCNE1 gene expression in MDB-MA-231 (ER-, PR-, HER2-), MCF7 (ER+, PR+, HER2-), BT474 (ER-, PR+, HER2+), and SkBr3 (ER-, PR-, HER2+) cells by qRT-PCR (*P < 0.05). Student’s *t*-tests were used to assess the statistical significance of differences

The expression levels of CCNE1 in MDB-MA-231 (ER-, PR-, HER2-), MCF7 (ER+, PR+, HER2-), BT474 (ER-, PR+, HER2+), and SkBr3 (ER-, PR-, HER2+) cells were detected by qRT-PCR. The results showed that the mRNA expression levels of CCNE1 were significantly higher in MDB-MA-231 (ER-, PR-, HER2-) than non-TNBC cells (Fig. 8, *P* < 0.05).

## Discussion

In order to recognize meaningful prognostic biomarkers of TNBC, this research analyzed two profile data sets (GSE36693 and GSE65216) using bioinformatic methods. A total of 76 TNBC specimens and 175 non-TNBC specimens were included in the current research. With analysis of GEO2R and Venn diagram, 140 common DEGs were identified (|logFC|>2 and adjusted *P*<0.01). Through KEGG pathway analysis, DEGs were obviously enriched in p53 signaling pathway, prostate cancer, and metabolic pathways (*P*<0.05). Then, by using the STRING online tool as well as Cytoscape, we constructed DEG PPI network complex composed of 94 nodes and 180 edges. In addition, 29 vital genes were screened from the PPI network complex via the analysis of Cytoscape’s MCODE. Next, we performed KEGG enrichment analysis again on the 29 DEGs through DAVID and found that these genes were enriched in the p53 signaling pathway, pathways in cancer, oocyte meiosis, and prostate cancer (*P*<0.05). Taking the results of both PPI analysis and the KEGG pathway enrichment into consideration, CCNE1, CDKN2A, AR, SERPINB5, and IGF1 of the 29 selected genes were found to play a key role in common significantly enriched pathways.

Three hub genes showed high expression with the validation of GEPIA expression sequencing. Finally, only CCNE1 had a significantly worse survival identified by survival analysis using cBioportal, which could be used as a new potential target for providing new treatment ideas for TNBC and improving prognosis. To further validate the prognostic signature, we conducted additional qRT-PCR analysis, which showed that the CCNE1 gene was significantly overexpressed in TNBC cells compared to non-TNBC cells (Fig. 8).

CCNE1, also known as cyclin E1, encoded by this gene belongs to the highly conserved cyclin family. The characteristic of its members is that the protein concentration changes drastically with the cycle throughout the cell cycle. Cyclin is a regulator of cyclin-dependent kinase (CDK); CCNE1 forms a complex with CDK2 as a regulatory subunit whose activity is necessary for cell cycle G1/S transition [20]. The protein is abundantly present at the boundary of G1-S phase and degraded as the cell cycle passes through S phase. This gene has been observed to be highly expressed in many tumors, which can lead to chromosomal instability and contribute to tumorigenesis. The dysregulation of CCNE1-CDK2 activity is related to a variety of cancers including nasopharyngeal carcinoma, bladder cancer, and breast cancer, and has been fully proven [21–23]. Accumulating data proved that TNBC frequently expressed CCNE1, while ER-positive cancer did not [24], and the absence of CCNE1 for poorer DFS [24].

In the p53 pathway, p53 acted as a tumor suppressor gene. Contrary to the activation of p53 regulatory checkpoint or apoptosis, the expression of cyclin E protein promotes the process of entering S phase from G1 phase. And the lack of p53 function gives tumor cells an escape gap, so that tumor cells can avoid cell cycle arrest or cell death and advance to the next stage through this disorder and uncontrolled growth [20, 25]. In addition, the loss of functional expression of the G1 checkpoint CDK inhibitor, p21, is also related to the carcinogenesis and disease progression of breast cancer; at the same time, more and more data indicate that the loss of function of p21 can mediate the drug-resistant phenotype which always means a poor prognosis [26]. In our bioinformatic analysis, the phenomenon of the upregulated CCNE1 enriched in the p53 pathway was verified again, and CCNE1 really plays an important role in TNBC, but relative clinical practice is lacking.

As for pathways in cancer, specifically in the cell cycle, the transcription factor E2F1 and the tumor suppressor protein retinoblastoma (RB) are two key factors that regulate the progression of the cell cycle. They determine whether the cell can carry out the process of DNA replication and cell division by regulating the checkpoints of G1/S and G2/M together [27]. The CCNE1/CDK2 complex can phosphorylate RB, then release E2F1 and activate its transcriptional activity to advance the cell cycle from G1 to S phase, while dephosphorylation of RB promotes E2F1 heterodimerization while inhibiting E2F1 activity [28, 29]. It can be seen in Fig. 7b that Cyclin E-CDK2 is associated with RB gene in the process of pathways in cancer, and this enrichment of CCNE1 was validated by us as well. There is no doubt that CCNE1 can be a useful target of TNBC in the future.

Numerous studies have proved that CCNE1 was related to the carcinogenesis and development of various types of cancer. Nevertheless, researches and clinical practice reported about this gene in TNBC is insufficient; in other words, they have not been taken seriously enough. Further experiments should be carried out on CCNE1 to better validate its functions. Thus, results in our research may provide useful information and directions for prospective research on TNBC treatment and prognosis. But this needs to be further verified through experiments in vitro or in vivo.

## Conclusions

CCNE1 could lead to a poorer prognosis in TNBC identified via bioinformatic analysis and plays a key role in the progression of TNBC which may contribute potential targets for the diagnosis, treatment, and prognosis assessment of TNBC.

## Supplementary Information

**Additional file 1: Supplementary Table 1**. Primer sequences used to amplify target genes in TNBC and non-TNBC cells by qRT-PCR.

**Additional file 2: Supplementary Table 2**. Gene Ontology analysis of differentially expressed genes in TNBC.

**Additional file 3: Supplementary Table 3**. KEGG pathway analysis of differentially expressed genes in TNBC.

**Additional file 4: Supplementary Table 4**. Re-analysis of 29 selected genes via Gene Ontology enrichment.

**Additional file 5: Supplementary Table 5**. Re-analysis of 29 selected genes via KEGG pathway enrichment.

## Data Availability

All the data in this research are available from GEO database and TCGA database.

## Abbreviations

TNBC: Triple-negative breast cancer
HER2: Human epidermal growth factor receptor 2
DEGs: Differentially expressed genes
HR: Homologous recombination
GEO: Gene Expression Omnibus
GO: Gene Ontology
KEGG: Kyoto Encyclopedia of Genes and Genomes
BP: Biological process
CC: Cellular component
MF: Molecular function
PPI: Protein-protein interaction
MCODE: Molecular Complex Detection
GEPIA: Gene Expression Profiling Interactive Analysis
OS: Overall survival
DFS: Distant free survival
CDK: Cyclin-dependent kinase
