# Supplementary material for: Overexpression of CCNE1 confers a poorer prognosis in triple-negative breast cancer identified by bioinformatic analysis
Source: World J Surg Oncol. 2021 Mar 23;19:86. doi: 10.1186/s12957-021-02200-x (PMC7989008; doi:10.1186/s12957-021-02200-x)
Supplement: Supplementary file 1 — Additional file 1: Supplementary Table 1. Primer sequences used to amplify target genes in TNBC and non-TNBC cells by qRT-PCR. [file 12957_2021_2200_MOESM1_ESM.doc]

| Supplementary Table 1. Primer sequences used to amplify target genes in TNBC and non-TNBC cells by qRT-PCR. | | |
| --- | --- | --- |
| Target Gene | Primer Sequence (5′-3′) | Primer information |
| β-Actin |  | NM_001101 |
| forward | GTCCACCGCAAATGCTTCTA |  |
| reverse | TGCTGTCACCTTCACCGTTC |  |
| CCNE1 |  | NM_001238.4 |
| forward | TAGAGAGGAAGTCTGGAAAATCATG |  |
| reverse | ATATACCGGTCAAAGAAATCTTGTG |  |
| TNBC: triple-negative breast cancer;  qRT-PCR: quantitative Real time- polymerase chain reaction. | | |
