# Supplementary material for: Overexpression of CCNE1 confers a poorer prognosis in triple-negative breast cancer identified by bioinformatic analysis
Source: World J Surg Oncol. 2021 Mar 23;19:86. doi: 10.1186/s12957-021-02200-x (PMC7989008; doi:10.1186/s12957-021-02200-x)
Supplement: Supplementary file 2 — Additional file 2: Supplementary Table 2. Gene Ontology analysis of differentially expressed genes in TNBC. [file 12957_2021_2200_MOESM2_ESM.doc]

| **Supplementary Table 2. Gene Ontology analysis of differentially expressed genes in TNBC.** | | | | | |
| --- | --- | --- | --- | --- | --- |
| Expression | Category | Term | Count | % | P Value |
| Up-regulated | GOTERM_BP_DIRECT | GO:0007422~peripheral nervous system development | 3 | 4.35 | 0.0036254 |
|  | GOTERM_BP_DIRECT | GO:0008544~epidermis development | 4 | 5.80 | 0.003993946 |
|  | GOTERM_BP_DIRECT | GO:0016337~single organismal cell-cell adhesion | 4 | 5.80 | 0.006458971 |
|  | GOTERM_CC_DIRECT | GO:0005615~extracellular space | 16 | 23.19 | 7.34E-05 |
|  | GOTERM_CC_DIRECT | GO:0005882~intermediate filament | 4 | 5.80 | 0.008000418 |
|  | GOTERM_CC_DIRECT | GO:0070062~extracellular exosome | 19 | 27.54 | 0.009474606 |
|  | GOTERM_CC_DIRECT | GO:0097209~epidermal lamellar body | 2 | 2.90 | 0.014409065 |
|  | GOTERM_MF_DIRECT | GO:0004568~chitinase activity | 2 | 2.90 | 0.024620645 |
|  | GOTERM_MF_DIRECT | GO:0008061~chitin binding | 2 | 2.90 | 0.028088866 |
| Down  -regulated | GOTERM_BP_DIRECT | GO:0014065~phosphatidylinositol 3-kinase signaling | 3 | 0.03 | 0.004160756 |
|  | GOTERM_BP_DIRECT | GO:0045944~positive regulation of transcription from RNA polymerase II promoter | 10 | 0.10 | 0.00769136 |
|  | GOTERM_CC_DIRECT | GO:0005615~extracellular space | 12 | 0.12 | 0.007648324 |
|  | GOTERM_MF_DIRECT | GO:0001085~RNA polymerase II transcription factor binding | 3 | 0.03 | 0.012119346 |
|  | GOTERM_MF_DIRECT | GO:0005509~calcium ion binding | 8 | 0.08 | 0.013256787 |
|  | GOTERM_MF_DIRECT | GO:0034056~estrogen response element binding | 2 | 0.02 | 0.014142799 |
| TNBC: triple-negative breast cancer. | | | | | |
