# Supplementary material for: Overexpression of CCNE1 confers a poorer prognosis in triple-negative breast cancer identified by bioinformatic analysis
Source: World J Surg Oncol. 2021 Mar 23;19:86. doi: 10.1186/s12957-021-02200-x (PMC7989008; doi:10.1186/s12957-021-02200-x)
Supplement: Supplementary file 3 — Additional file 3: Supplementary Table 3. KEGG pathway analysis of differentially expressed genes in TNBC. [file 12957_2021_2200_MOESM3_ESM.doc]

| **Supplementary Table 3. KEGG pathway analysis of differentially expressed genes in TNBC.** | | | | |
| --- | --- | --- | --- | --- |
| Category | Term | Count | % | P Value |
| KEGG_PATHWAY | hsa04115: p53 signaling pathway | 4 | 2.88 | 0.008797166 |
| KEGG_PATHWAY | hsa05215: Prostate cancer | 4 | 2.88 | 0.018367061 |
| KEGG_PATHWAY | hsa01100: Metabolic pathways | 14 | 10.07 | 0.037159241 |
| TNBC: triple-negative breast cancer. | | | | |
