# Supplementary material for: Overexpression of CCNE1 confers a poorer prognosis in triple-negative breast cancer identified by bioinformatic analysis
Source: World J Surg Oncol. 2021 Mar 23;19:86. doi: 10.1186/s12957-021-02200-x (PMC7989008; doi:10.1186/s12957-021-02200-x)
Supplement: Supplementary file 4 — Additional file 4: Supplementary Table 4. Re-analysis of 29 selected genes via Gene Ontology enrichment. [file 12957_2021_2200_MOESM4_ESM.doc]

| **Supplementary Table 4. Re-analysis of 29 selected genes via Gene Ontology enrichment.** | | | |
| --- | --- | --- | --- |
| Category | Term | Count | P Value |
| GOTERM_BP_DIRECT | GO:0045893~positive regulation of transcription, DNA-templated | 9 | 9.84E-07 |
| GOTERM_BP_DIRECT | GO:0045944~positive regulation of transcription from RNA polymerase II promoter | 11 | 1.50E-06 |
| GOTERM_BP_DIRECT | GO:0006366~transcription from RNA polymerase II promoter | 7 | 1.35E-04 |
| GOTERM_BP_DIRECT | GO:0048546~digestive tract morphogenesis | 3 | 1.92E-04 |
| GOTERM_BP_DIRECT | GO:0043568~positive regulation of insulin-like growth factor receptor signaling pathway | 3 | 1.92E-04 |
| GOTERM_BP_DIRECT | GO:0014065~phosphatidylinositol 3-kinase signaling | 3 | 8.52E-04 |
| GOTERM_CC_DIRECT | GO:0005882~intermediate filament | 4 | 6.79E-04 |
| GOTERM_CC_DIRECT | GO:0005615~extracellular space | 9 | 7.11E-04 |
| GOTERM_CC_DIRECT | GO:0005634~nucleus | 16 | 0.006994347 |
| GOTERM_CC_DIRECT | GO:0070062~extracellular exosome | 10 | 0.020917677 |
| GOTERM_CC_DIRECT | GO:0000790~nuclear chromatin | 3 | 0.035216711 |
| GOTERM_CC_DIRECT | GO:0030057~desmosome | 2 | 0.036252678 |
| GOTERM_MF_DIRECT | GO: 0008134~transcription factor binding | 7 | 5.93E-06 |
| GOTERM_MF_DIRECT | GO: 0043565~sequence-specific DNA binding | 7 | 1.72E-04 |
| GOTERM_MF_DIRECT | GO: 0000981~RNA polymerase II transcription factor activity, sequence-specific DNA binding | 5 | 1.72E-04 |
| GOTERM_MF_DIRECT | GO: 0044212~transcription regulatory region DNA binding | 5 | 3.98E-04 |
| GOTERM_MF_DIRECT | GO: 0005515~protein binding | 24 | 9.40E-04 |
| GOTERM_MF_DIRECT | GO: 0001085~RNA polymerase II transcription factor binding | 3 | 0.002738639 |
| TNBC: triple-negative breast cancer. | | | |
