# Supplementary material for: Overexpression of CCNE1 confers a poorer prognosis in triple-negative breast cancer identified by bioinformatic analysis
Source: World J Surg Oncol. 2021 Mar 23;19:86. doi: 10.1186/s12957-021-02200-x (PMC7989008; doi:10.1186/s12957-021-02200-x)
Supplement: Supplementary file 5 — Additional file 5: Supplementary Table 5. Re-analysis of 29 selected genes via KEGG pathway enrichment. [file 12957_2021_2200_MOESM5_ESM.doc]

| **Supplementary Table 5 . Re-analysis of 29 selected genes via KEGG pathway enrichment.** | | | | |
| --- | --- | --- | --- | --- |
| Category | Term | Count | P Value | Genes |
| KEGG_PATHWAY | hsa04115: p53 signaling pathway | 4 | 1.38E-04 | CCNE1, CDKN2A, SERPINB5, IGF1 |
| KEGG_PATHWAY | hsa05215:  Prostate cancer | 3 | 0.008256173 | CCNE1, AR, IGF1 |
| KEGG_PATHWAY | hsa04114:  Oocyte meiosis | 3 | 0.012905535 | CCNE1, AR, IGF1 |
| KEGG_PATHWAY | hsa05200:  Pathways in cancer | 4 | 0.021651652 | CCNE1, AR, CDKN2A, IGF1 |
| TNBC: triple-negative breast cancer. | | | | |
